# Bone resorption and environmental exposure to cadmium in children: a cross - sectional study

**DOI:** 10.1186/1476-069X-10-104

**Published:** 2011-12-08

**Authors:** Muhammad Sughis, Joris Penders, Vincent Haufroid, Benoit Nemery, Tim S Nawrot

**Affiliations:** 1 Department of Public Health, Occupational and Environmental Medicine, Unit of Lung Toxicology, Katholieke Universiteit Leuven, Leuven, Belgium; 2 Centre of Research for Public Health, Lahore, Pakistan; 3 Lahore College of Pharmaceutical Sciences, Lahore, Pakistan; 4 Biomedical Research Institute, Hasselt University, Diepenbeek, Belgium; 5 Department of Clinical Chemistry, Microbiology and Immunology, Ghent University Hospital, Ghent, Belgium; 6 Laboratory of the Industrial Toxicology and Occupational Medicine Unit (Université Catholique de Louvain, Belgium), Brussels, Belgium; 7 Centre for Environmental Sciences, Hasselt University, Diepenbeek, Belgium

**Keywords:** Children, Calcium, Deoxypyridinoline, Environmental Cadmium Exposure, Osteoporosis

## Abstract

**Background:**

Exposure to cadmium has been associated with osteoporosis and fracture risk in women and elderly, but studies in children are lacking. In the present study we investigate the association between markers of bone demineralization [urinary calcium (Ca) and deoxypyridinoline (DPD) excretion] and urinary cadmium (Cd) excretion (as an index of lifetime body burden).

**Methods:**

155 schoolchildren from 2 elementary schools in Lahore, Pakistan were included. Urinary Cd was measured as an index of lifetime exposure. We assessed the multivariate-adjusted association of exposure with markers of bone resorption, urinary DPD as well as with Ca excretion.

**Results:**

Urinary Cd averaged 0.50 nmol/mmol creatinine and was not influenced by age, height, weight and socio-economic status (SES). Independent of gender, age, height, weight and SES a doubling of urinary Cd was associated with a 1.72 times (p < 0.0001) increase in urinary DPD and, a 1.21 times (p = 0.02) increase in urinary Ca. Additional adjustment for urinary Ca revealed still significant associations between urinary Cd and urinary DPD. The shape of the association was linear without evidence of a threshold.

**Conclusions:**

Even in young children, low-level environmental exposure to cadmium is associated with evidence of bone resorption, suggesting a direct osteotoxic effect with increased calciuria. These findings might have clinical relevance at older age.

## Background

Cadmium (Cd) is a heavy metal that is widely present in our environment as a pollutant [1]. The environmental presence of Cd is attributed to industrial emissions and agriculture [2]. Populations worldwide are exposed to Cd by low-level intake mainly through their food or by inhalation of tobacco smoke and exposure to Cd contaminated airborne particles. Presence of Cd in the atmosphere due to industrial activities and the use of fertilizers containing Cd may lead to the contamination of land and increased uptake of Cd by crops and vegetables cultivated for human consumption [2]. Dust is potentially an important route of exposure to heavy metals in areas with contaminated soils [3]. Environmental measures in Lahore, Pakistan showed annual mean fine particalute (PM_10_) Cd concentration of 77 ng/m^3^(vs 5 ng/m^3 ^WHO guidelines) [4]. Exposure to Cd leads to an age-related cumulative increase in the body burden of this toxic metal [5]. The urinary Cd concentration reflects life-time exposure [6]. Cd causes tubular renal dysfunction [7] and increases calciuria [8].

Clinically, osteoporosis is a disease of middle to old age; risk factors operating in early life have been barely investigated. Clinical features associated with osteoporosis include increased morbidity (i.e. pain, physical impairment, and decreased quality of life), increased risk of fractures, and increased mortality [9]. Studies among populations from Belgium [10,11], Sweden [12-14], Japan [15], and China [16,17] showed associations between osteoporosis and low-level environmental Cd exposure. The generally accepted interpretation has been that Cd induces renal tubular damage, decreases the Ca reabsorption in the nephron, thus resulting in hypercalciuria [7] and low bone mineral density (BMD) and hence increased fracture risk [10,15] particularly in postmenopausal women [10,11] or men in the older age group [15]. However, a recent study also found a dose-response association between risks of osteoporosis in middle aged men (mean age 45 y) and urinary Cd [18]. Independently of the status of kidney function, other data also support a direct osteotoxic effect of Cd, on the basis of an increase in the urinary excretion of pyridinium crosslinks from bone collagen even at low non-nephrotoxic concentrations of urinary Cd in postmenopausal women [11].

Deoxypyridinoline (DPD) cross links occur in Type I collagen of bone and results by the action of lysyl oxidase on two hydroxylysine and a lysine residue. Any process which may lead to the bone degradation, leads to the release of DPD into the blood circulation and cleared by kidneys. Hence DPD has been shown to be a biochemical indicator of bone resorption. In view of the omnipresence distribution of Cd pollution [19] and epidemic of osteotoxicity [20], we measured urinary pyridinium crosslinks of collagen, which is a specific marker of bone resorption [21] to investigate the possible direct osteotoxicity of Cd beyond its indirect effect on bone via increased calciuria [8] in schoolchildren living in a metropolitan city of Pakistan.

## Methods

### Study areas and subject recruitment

The study subjects were 8 to 12 year old children attending two private elementary schools in Lahore, Pakistan. The study was conducted between January and April 2009 in accordance with the ethical standards according to the Helsinki Declaration and was approved by Lahore College of Pharmaceutical Sciences, Lahore, Pakistan. Details of this environmental health study in school children of Pakistan have been published (Muhammad Sughis, Tim S Nawrot, Syed Ihsan-ul-Haque, Asad Amjad and Benoit Nemery: Blood pressure and particulate air pollution in schoolchildren of Lahore, Pakistan, submitted). Briefly, the school officials were first contacted through private contacts. The school officials then communicated with the parents seeking their consent for their children to participate in the study. The sole inclusion criteria to participate in the study were the prescribed age range, verbal approval of parents and willingness of the participant. The participants received verbal and written information about the purpose of the study. Of the 192 children that were asked to participate were explained the objectives, procedure and the duration of the examination. 179 (94%) gave written informed consent. Urine samples were collected from 173 participants of whom 155 had urine measurements for DPD. We coded social economic status based on education of the father and condensed it into a scale with scores ranging from 1 to 3. Urinary Ca, DPD and Cd are the part of larger set of endpoints including blood pressure measurements, respiratory health (spirometry and exhaled NO), and estimation of 20 (Be, Al, V, Cr, Mn, Co, Ni Cu, Zn, As, Se, Mo, Cd, Sn, Sb, Ba, Tl, Pb, Bi and U) toxic metals in urine including Cd. This article focus on the association between Ca and DPD excretion and Cd exposure, as Cd bone effects have been described in adult populations [13,18,22], but so far these have not been studied in children.

### Measurements in urine

A spot urine sample was collected between 9:00 am and 2:00 pm using a sterile polystyrene container (100 ml). To avoid contamination of the sample, the children were asked to wash hands before and after sampling. Creatinine was determined on the day of sampling using a kit from DiaSys Diagnostic Systems, Germany as per instructions from the manufacturer. Aliquots of urine were transferred in eppendorf tubes in triplicates with disposable dropper and kept frozen. Later the samples were transported on dry ice (at -80°C) to Belgium for further analysis. The urine sample was analyzed in the Laboratory of the Industrial Toxicology and Occupational Medicine Unit (Université catholique de Louvain, Belgium) without knowledge of their exact provenance in relation to exposure (blind analysis). In all urine samples, the concentration of 20 metals or metalloids (all called "metals" hereafter) were quantified by means of inductively coupled argon plasma mass spectrometry (ICP-MS) with an Agilent 7500 ce instrument. Briefly, urine specimens (500 μl) were diluted quantitatively (1+9) with a HNO_3 _1%, HCl 0.5% solution containing Sc, Ge, Rh and Ir as internal standards. Ba, Sb, Al, Cd, Pb, Mo, Sn, Be, Tl, Bi and U were analyzed using no gas mode while helium mode was selected to quantify As, V, Cr, Mn, Co, Ni, Cu, Zn, and Se. Using this validated method, the laboratory has obtained successful results in external quality assessment schemes organized by the Institute for Occupational, Environmental and Social Medicine of the University of Erlangen, Germany (G-EQUAS program), and by the Institut de National Santé Publique, Québec (PCI and QMEQUAS programs). The limit of detection (LOD) and limit of quantification (LOQ) for Cd was 0.015 μg/l and 0.044 μg/l respectively. The inter assay coefficient of variance was < 3% for certified internal control (Recipe, ClinCheck control level I and II).

Urinary Ca was analyzed using an automated photometric analyzer (Modular^® ^P800-ISE900 System, Roche Diagnostics; Mannheim, Germany) at the Clinical Laboratory of Ziekenhuis Oost-Limburg, Genk, Belgium. The urine samples were not acidified by EDTA. Free DPD was measured in spot urine samples by ELISA (Cusabio Biotech, China). For DPD analysis, the inter-assay coefficient of variation for duplicate samples was 8% (n = 155).

### Statistical analysis

We used SAS software version 9.2 (SAS Institute Inc, Cary, NC) and GraphPad Prism version 4.2. Non-normally distributed data were logarithmically transformed and presented as geometric mean (5^th ^to 95^th ^percentile). For comparison of means and proportions, we applied Student's t-test and the χ^2^-statistic, respectively. We plotted the biomarkers of effect and urinary Cd to ensure that there was no threshold phenomenon and that linear correlation techniques were appropriate. We investigated associations between biomarkers of effect and exposure using single and multiple linear regressions. Covariates considered for adjustment in the model included gender, age, height, weight, and socioeconomic class. In addition, in the sensitivity analysis, time of the day of the urine collection was considered. Potential interactions between Cd and gender on urinary levels of DPD and Ca were investigated. Q-Q plots of the residuals were used to test the assumptions of all linear models. The urinary Ca and DPD were not normally distributed and therefore logarithmically transformed. We retransformed to linear scale and expressed effects as percent change for a two-fold increase in urinary Cd.

## Results

Children with missing values for urinary DPD were excluded from the analysis (n = 18). The characteristics of the 155 children are listed in Table 1. Urinary DPD (216 nmol/mmol creatinine *vs *83.5 nmol/mmol creatinine; p < 0.0001) and Cd (0.65 nmol/mmol *vs *0.41 nmol/mmol creatinine; p < 0.0001) concentrations were higher in girls compared with boys. Urinary DPD concentration did not correlate with age, height or weight.

**Table 1:** Characteristics of participants from a metropolitan city of Pakistan

Characteristics	Boys (n = 87)	Girls (n = 68)	School Children (n = 155)
Age - yr	9.9 (1.3)	9.6 (1.2)	9.8 (1.2)

Height - cm	134 (9.8)	132 (9.0)	133 (9.5)

Weight - kg	28.8 (7.2)	27.0 (6.4)	28.0 (6.9)

BMI - kg/m^2^	15.9 (2.9)	15.3 (2.2)	15.7 (2.6)

Creatinine - mmol/l	11.8 (7.0)	7.4 (3.5)	9.9 (6.1)

Cadmium - nmol/mmol crt	0.41 (0.15 to 1.58)	0.65 (0.20 to 1.63)	0.50 (0.16 to 1.63)

Calcium - μmol/mmol crt	71.0 (13.3 to 401)	108 (16.9 to 494)	85.3 (15.7 to 451)

Deoxypyridinoline - nmol/mmol crt	83.5 (12.5 to 695)	216 (21.8 to 3502)	127 (14.6 to 1945)

Data are arithmetic mean (SD) or geometric mean (5^th ^to 95^th ^percentile).

**Figure 1:**
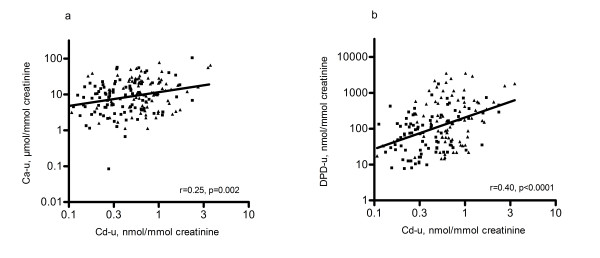
**Association of urinary Ca (a) and DPD (b) with Cd**. Association of Ca (a) and DPD (b) in urine with urinary concentration of Cd. The square data points represent boys and triangle represents girls.

Both before (Figure 1a &1b) and after adjustment for gender, age, height, weight and socioeconomic class, the concentration of urinary DPD and Ca increased significantly with higher urinary Cd (Table 2). In secondary analysis, to gain more insight in the direct effects of Cd on bone, we studied the association between urinary excretion of DPD and Cd, correcting for urinary Ca. The urinary DPD concentration did not correlate significantly (p = 0.6) with urinary Ca. Along with the previous mentioned covariates, we added urinary Ca to the regression model in which we predicted urinary DPD by urinary Cd: each doubling of urinary Cd was associated with 1.72 times increase in urinary DPD (95% CI: 1.37 to 2.17; *p: *< 0.0001). We tested the gender by Cd interaction on urinary levels of DPD and Ca but no effect-modification by gender was observed (p ≥ 0.3). Nevertheless, the gender stratified results have been given in Table 2, showing a consistent pattern of urinary markers of bone and cadmium in girls and boys. In addition, we run sensitivity analysis with additional adjustment for the time of the day of urine collection as a marker of diurnal variation. This additional adjustment did not alter our previous mentioned association.

**Table 2:** Percent change in urinary deoxypyridinoline (DPD) and calcium (Ca) for doubling in urinary Cd concentration

Biomarkers of bone resorption	Boys (n = 87)		Girls (n = 68)		**Schoolchildren (n = 155)**^**a**^	
	Effect Size* (95% CI)	**p-value**	Effect Size* (95% CI)	**p-value**	Effect Size* (95% CI)	**p-value**

Deoxypyridinoline, nmol/mmol crt^b^	71 (33 - 121)	< 0.0001	86 (20 - 188)	0.007	72 (37 - 117)	< 0.0001

Deoxypyridinoline, nmol/mmol crt^c^	80 (38 - 133)	< 0.0001	84 (18 - 186)	0.008	74 (38 - 121)	< 0.0001

Calcium, μmol/mmol crt^b^	29 (3.0 - 61)	0.02	9.0 (-19 - 47)	0.5	21 (2.0 - 44)	0.02

Calcium, μmol/mmol crt^d^	39 (9.0 - 77)	0.009	6.0 (-23 - 45)	0.7	24 (3.0 - 50)	0.02

*Regression coefficients (95% confidence [CI]) have been calculated for a doubling in the urinary cadmium concentration and expressed as % increase.

**^a ^**gender by Cd interaction on urinary levels of DPD and Ca indicated no effect-modification by gender (p for interaction ≥0.3).

^b ^adjusted for gender, age, height, weight and socioeconomic status.

^c ^adjusted for calcium, gender, age, height, weight and socioeconomic status.

^d ^adjusted for DPD, gender, age, height, weight and socioeconomic status.

## Discussion

Cd is stored in the kidney from birth. Therefore, its urinary excretion shows life-time exposure [19]. We found that urinary Cd is associated with higher urinary Ca and DPD excretion, markers of bone demineralization and bone resorption in children, suggesting that the skeletal demineralization effects of Cd occur, a) at relatively low environmental concentrations, b) start early in life.

Experimental studies strongly support the epidemiologic evidence for a direct osteotoxic effect of Cd. In animals exposed to Cd, bone demineralization begins early after the start of Cd exposure and well before the onset of kidney damage [23,24]. The urinary Cd concentration in our population averaged 0.53 nmol/mmol creatinine. This is comparable with other studies in Pakistan [25] and considerable higher than data from Europe (0.14 nmol/mmol creatinine) [26] or of the US NHANES population showing urinary Cd values in children of 0.07 nmol/l [27].

Exposure to Cd occurs through intake of contaminated food or water. In exposed populations, house dust loaded with cadmium is an additional relevant exposure route [3]. In addition recently, potential for dangerous Cd exposure has been suggested to children who wear, mouth, or accidentally swallow on inexpensive jewelry which often contains high levels of Cd (10 000 ppm) [28]. In our study area, relevant air concentrations of Cd have been reported (14 times higher than the WHO guideline) [4].

Our findings might have important implications for environmental policies, especially those designed to protect children's health and prevention of factors that are operative early in life as shown here. At middle-age, osteoporosis occurs at an accelerated rate. The studies in Belgium [10], China [16] and Sweden [22] demonstrated loss of BMD in relation to Cd exposure, which was more severe in women [11,16], particularly after the onset of menopause [11].

In the adult population, women have a higher body burden of Cd than men [29]. In line with these observations in adults, in our sample girls had considerably higher body Cd compared with boys. Children take up Cd more readily than adults due to lower iron stores [5,30,31]. The Cd iron interaction is a consequence of upregulation of iron transporter divalent metal transporter-1 in the intestine at low iron concentrations. After upregulation of metal transporter-1 there is a greater potential for increased absorption of Cd [32]. The higher Cd stores in girls compared with boys, did not confound our reported associations as our analysis was consistently observed in models stratified by gender.

The present study has limitations and strengths. Although our results were consistent after multiple adjustments and in sensitivity analyses, we cannot exclude residual confounding. In most circumstances, bone collagen degradation is the major contributor to both crosslink compounds in urine, due to the low turnover rate of other tissues [21]. The urinary excretion of bone collagen has a diurnal variation, with levels peaking in the morning [33]. Additional adjustments for time of the day of urine collection exclude potential confounding by diurnal variations. Finally, our urine samples were not acidified by EDTA; therefore, Ca precipitation might have been occurred. However, Ca precipitation is proportional to the urinary Ca concentration and it is unlikely that this has biased our results.

## Conclusions

We found a consistent association between biomarkers of bone resorption and bone demineralization and Cd exposure in 10-year old children. These findings might have clinical consequences in adult life. Because Cd is a long-lived multi-organ toxicant that remains in a child's body into adulthood, Cd exposure must be limited as much as possible from early age on.

## List of abbreviations

Ca: calcium; DPD: deoxypyridinoline; Cd: cadmium; BMD: bone mineral density; PM: particulate matter; ICP-MS: inductively coupled plasma mass spectrometry; LOD: limit of detection; LOQ: limit of quantification; SES: socio-economic status.

## Competing interests

The authors declare that they have no competing interests.

## Authors' contributions

MS, TSN and BN designed the study. MS collected urine samples. MS measured urinary DPD, JP measured urinary Ca and MS and VH analysed urinary Cd. MS, TSN, and BN contributed in the discussion of data, drawn conclusions, and drafted the manuscript. All authors read and approved the final manuscript.
